# Changes in the gut microbiota structure and function in rats with doxorubicin-induced heart failure

**DOI:** 10.3389/fcimb.2023.1135428

**Published:** 2023-04-27

**Authors:** Yawen Fan, Lichang Liang, Xinzheng Tang, Jinxian Zhu, Lei Mu, Mengni Wang, Xuecheng Huang, Shenglan Gong, Jinghan Xu, Tianjiao Liu, Tianfeng Zhang

**Affiliations:** ^1^ Department of Cardiovascular Diseases, The Sixth Clinical Medical School of Guangzhou University of Chinese Medicine, Shenzhen Hospital of Guangzhou University of Chinese Medicine (Futian), Shenzhen, China; ^2^ Department of Preventive Treatment, The Sixth Clinical Medical School of Guangzhou University of Chinese Medicine, Shenzhen Hospital of Guangzhou University of Chinese Medicine (Futian), Shenzhen, China; ^3^ Department of Encephalopathy Diseases, Shenzhen Hospital of Beijing University of Chinese Medicine (Longgang), Shenzhen, China; ^4^ Department of Spinal Surgery, The Sixth Clinical Medical School of Guangzhou University of Chinese Medicine, Shenzhen Hospital of Guangzhou University of Chinese Medicine (Futian), Shenzhen, China; ^5^ Department of Endocrinology, The Sixth Clinical Medical School of Guangzhou University of Chinese Medicine, Shenzhen Hospital of Guangzhou University of Chinese Medicine (Futian), Shenzhen, China

**Keywords:** doxorubicin, heart failure, gut microbiota, 16S rRNA gene sequencing, intestinal hypothesis, animal model

## Abstract

**Objectives:**

The rat model of heart failure (HF) induced by doxorubicin (DOX), a broad spectrum and highly effective chemotherapeutic anthracycline with high-affinity to myocardial tissue that causes severe dose-dependent irreversible cardiotoxicity has been widely recognized and applied in HF pathogenesis and drug therapy studies. The gut microbiota (GM) has attracted significant attention due to its potential role in HF, and research in this area may provide beneficial therapeutic strategies for HF. Considering the differences in the route, mode, and total cumulative dose of DOX administration used to establish HF models, the optimal scheme for studying the correlation between GM and HF pathogenesis remains to be determined. Therefore, focusing on establishing the optimal scheme, we evaluated the correlation between GM composition/function and DOX-induced cardiotoxicity (DIC).

**Methods:**

Three schemes were investigated: DOX (at total cumulative doses of 12, 15 or 18 mg/kg using a fixed or alternating dose via a tail vein or intraperitoneal injection) was administered to Sprague Dawley (SD) for six consecutive weeks. The M-mode echocardiograms performed cardiac function evaluation. Pathological changes in the intestine were observed by H&E staining and in the heart by Masson staining. The serum levels of N-terminal pre-B-type natriuretic peptide (NT-proBNP) and cardiac troponin I (cTnI) were measured by ELISA. The GM was analysed by 16S rRNA gene sequencing.

**Key findings:**

Strikingly, based on the severity of cardiac dysfunction, there were marked differences in the abundance and grouping of GM under different schemes. The HF model established by tail vein injection of DOX (18 mg/kg, alternating doses) was more stable; moreover, the degree of myocardial injury and microbial composition were more consistent with the clinical manifestations of HF.

**Conclusions:**

The model of HF established by tail vein injection of doxorubicin, administered at 4mg/kg body weight (2mL/kg) at weeks 1, 3 and 5, and at 2mg/kg body weight (1mL/kg) at weeks 2, 4 and 6, with a cumulative total dose of 18mg/kg, is a better protocol to study the correlation between HF and GM.

## Introduction

Heart failure (HF) is the terminal stage of almost all kinds of heart disease, characterized by high morbidity, mortality, and readmission rates (Ziaeian and Fonarow, 2016; Roger, 2021). Despite new developments in modern comprehensive therapy strategies, the prognosis of HF remains bleak (Roger, 2021; Zhang et al., 2023). As a global healthcare epidemic, exploring the potential pathogenesis and determining new therapeutic targets for HF is vital. The gut microbiota (GM) constitutes the human intestinal micro-ecosystem and, together with its metabolites, such as short-chain fatty acids (SCFAs), trimethylamine-N-oxide (TMAO) and bile acid (BA), are involved in bodily metabolic processes such as signalling molecules and have emerged as central factors affecting human health and disease (Sonnenburg and Bäckhed, 2016; Kitai and Tang, 2018; Smolinska et al., 2018). There is considerable evidence that GM not only plays an essential role in the pathogenesis of HF but also is integral to the development of risk factors for HF, such as atherosclerosis, hypertension, diabetes and obesity (Jie et al., 2017; Metra and Teerlink, 2017; Kang and Cai, 2018; Lone et al., 2018). Therefore, GM has become a new hotspot in preventing and treating HF.

The researchers proposed the “gut hypothesis of HF “ (Nagatomo and Tang, 2015) based on the correlation between GM and HF. The theory states that patients with HF have impaired cardiac pump function, with reduced cardiac output leading to inadequate tissue perfusion and redistribution of the systemic blood supply. As the earliest organ to undergo ischaemia and hypoxia, ischaemia-reperfusion injury to the intestinal mucosa, as well as hypoxia to the villi and ends of the intestinal epithelium, leads to damage to the intestinal epithelium, intestinal barrier dysfunction and a marked increase in intestinal permeability (Kamo et al., 2017a). In addition, gastric mucosal carbon dioxide pressure rises in patients with HF, even when patients engage in low-level activities; weakened intestinal wall circulation and microcirculation disorders also lead to intestinal mucosal oedema and poor absorption, resulting in weakened immune defence function and ability to resist bacterial adhesion (Sandek et al., 2007; Li and Liu, 2021). At this time, enteric-derived bacterial endotoxins, such as lipopolysaccharide (LPS), an essential substance in maintaining the proinflammatory state in HF, can enter the blood circulation from the intestinal lumen through the damaged intestinal mucosa, causing the translocation of the GM and endotoxemia, initiating signal transduction pathways leading to activation and excessive release of inflammatory factors (such as tumour necrosis factor, interleukin, and C-reactive protein), which through the waterfall effect, promote the inflammatory response and exacerbate the HF process (Zabell and Tang, 2017; Mayerhofer et al., 2018; Chen et al., 2021; Hao et al., 2021; Lv et al., 2022). More and more research supports this hypothesis, but further verifications are still needed (Yoriko et al., 2017; Peng et al., 2018).

Mature and stable animal models play an role in investigations of mechanism of disease and the transformation from basic science to the clinic. Doxorubicin (DOX) induction widely used in the establishment of animal models of HF, and models can predict the formation time of HF according to the dose (Lu et al., 2018; Zhong et al., 2021). inconsistencies regarding the administration route, mode and cumulative dose DOX induction, success rate, mortality and quality In recent years, has been limited to changes in cardiac structure and function (Xu et al., 2016; Ye et al., 2016; Wang and Wang, 2017; Ma, 2019; Zhang et al., 2020; Cheng et al., 2011). However, in the GM across different DOX deserve attention.

The animal models, which are mature, stable and more similar to clinical practice, play an essential role in investigating the disease mechanism and the transformation from basic science to the clinic. Doxorubicin (DOX) induction has been widely used in the establishment of animal models of HF, and such models can predict the formation time of HF according to the dose (Lu et al., 2018; Zhong et al., 2021). Inconsistencies in DOX induction regarding the route, mode of administration and cumulative dose have resulted in models with varying success rates, mortality and quality. In recent years, assessment of DOX induction protocols has been mostly limited to changes in cardiac structure and function (Cheng et al., 2011; Xu et al., 2016; Ye et al., 2016; Wang and Wang, 2017; Ma, 2019; Zhang et al., 2020). However, as the knowledge of the correlation between GM and HF grows, the variation in GM across different DOX induction protocols deserves attention. Therefore, three protocols were designed to establish HF animal models in this study, including two routes of administration, two modes of administration and three total doses. In combination with 16S rRNA gene sequencing, the optimal protocol for investigating the correlation between HF and GM was determined by observing the changes in the composition and function of the GM.

## Material and methods

### Animal

Ninety-nine specific pathogen-free (SPF)-grade male Sprague Dawley (SD) rats aged 6–8 weeks (body weight: 200 ± 20 g) were purchased from Zhuhai BesTest Bio-Tech Co., Ltd., China (Zhuhai, China), license number: SCXK (Guangdong) 2020-0051. The experimental rats were housed in the SPF Laboratory of Shenzhen Top Biotechnology Co., Ltd., China (Shenzhen, China), license number: SYXK (Guangdong) 2020-0230. The rats were fed maintenance feed under a barrier system (free intake of water, 22-24°C, 12-hour light/dark cycle, relative humidity 67%, good ventilation).

**Figure 1:**
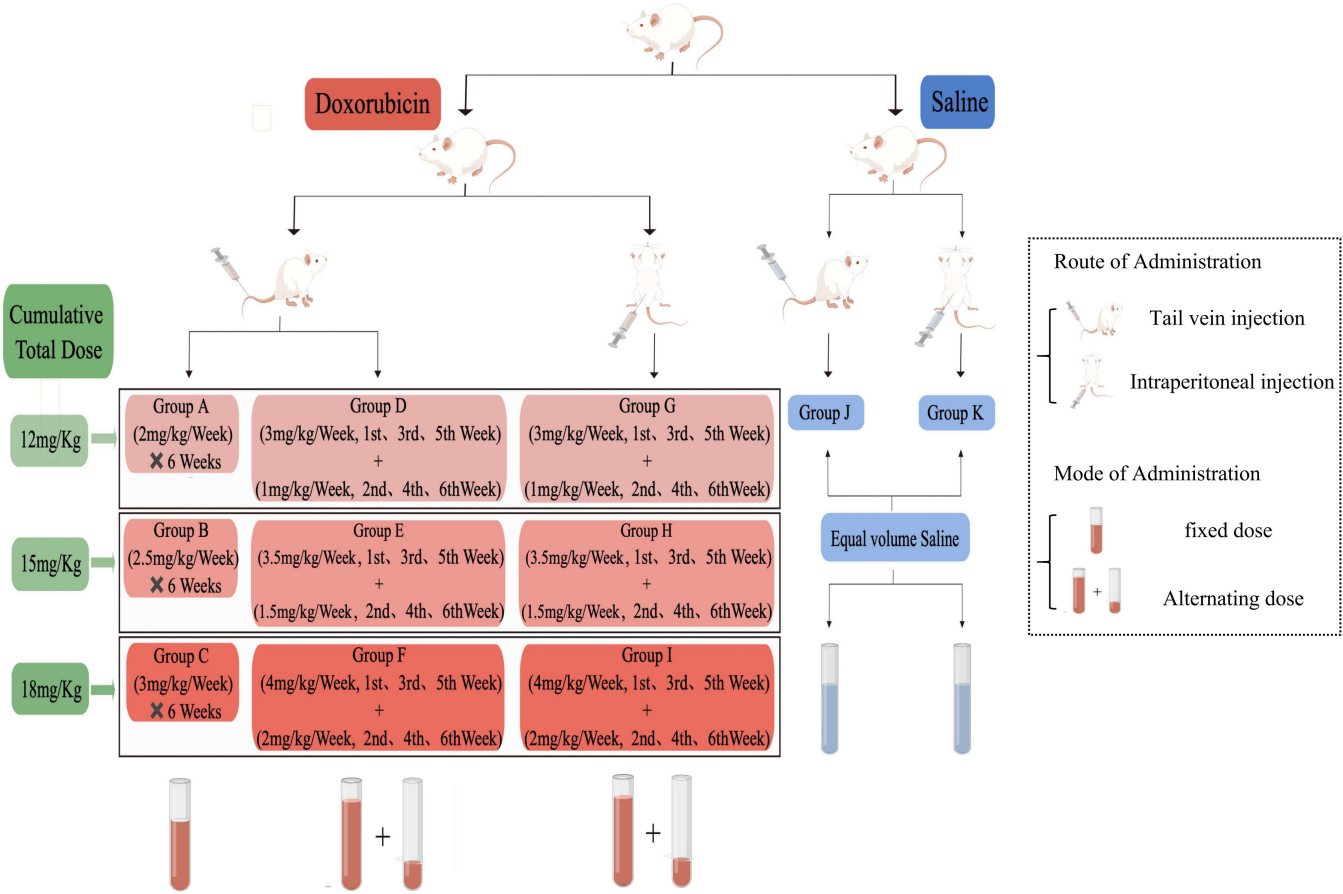
Schematic diagram of the study design.

All rats were adaptively fed for one week and randomly divided into 11 groups (n=9 rats per group), two of which were injected with normal saline. The remaining nine groups were injected with DOX (dissolved in 0.9% NaCl, Hanwai Pharmaceutical Co., Ltd, lot no. 21014711, Zhejiang, China). At the same time, each group was intervened once a week for six weeks and weighed regularly to determine the subsequent dose. The method of animal treatment to the control (CON) and DOX groups are displayed in **Figure 1**, which includes two routes of administration (tail vein injection(TVI) and intraperitoneal injection (II)), two modes of administration (fixed dose and alternating dose), and three total cumulative doses (12 mg/kg, 15 mg/kg and 18 mg/kg).

### Echocardiography assessment of cardiac function

Six weeks after the interventions, rats were fixed supine position after isoflurane (4% for induction and 1.8% for maintenance, Shenzhen Ruiwode Life Technology Co., Ltd., batch number: R510-22-16, Shenzhen, China) inhalation anaesthesia. Noninvasive transthoracic echocardiography was performed with a 13-MHz frequency wire probe near the sternum of the rats using an animal-specific Vevo 2100 high-resolution imaging system (Visual Sonics Inc., Toronto, Ontario, Canada). M-mode echocardiograms of the left ventricular short axis were captured at the level of the papillary muscle to determine the left ventricular internal diameter at end-diastole (LVID;d) and at end-systole (LVID;s), interventricular septal thickness at end-diastole (IVS;d) and at end-systole (IVS;s), left ventricular fractional shortening (LVFS), and left ventricular ejection fraction (LVEF). These parameters were obtained and averaged from the three cardiac cycles with n = 5/group.

### Blood collection and measurement of serum biochemical indices

At the end of the experiment, the rats in each group were fasted overnight without water deprivation for 12 hours, and their body weight (BW) was measured; then, after isoflurane inhalation anaesthesia, the rats were fixed in the supine position, and 5-8mL of blood from the abdominal aorta was drawn into centrifuge tubes. After standing at room temperature for 1 h, the collected blood samples were centrifuged at 4°C and 3000 rpm/min for 15 min to obtain serum, which was frozen at -80°C. The serum levels of the biochemical parameters, including N-terminal pro-B-type natriuretic peptide (NT-proBNP) and cardiac troponin I (cTnI), were measured with an enzyme-linked immunosorbent assay (ELISA) by using an NT-proBNP kit (Jiangsu Meimian Industrial Co., Ltd, China, catalog No: MM-0329R1) and cTnI kit (Jiangsu Meimian Industrial Co., Ltd, China, catalog No: MM-61550R1), according to the manufacturer’s protocols. Absorbance was measured for samples and standards at 450 nm using an enzyme labeling instrument (model 320, Labsystems Multiskan, MS, Finland).

### Histopathological analysis

After blood was taken, the thoracic cavity of the rats was completely exposed, the right atrial appendage was cut, and the abdominal aorta was immediately perfused with precooled phosphate-buffered saline (PBS). After perfusion, the heart and 2 cm of tissue in the middle region of the small intestine (ileum) were quickly removed and rinsed repeatedly with precooled 0.9% sodium chloride solution. The residual capsule and blood vessels were cut, dried with filter paper, and weighed with an electronic balance. The heart weight (HW) to body weight (BW) ratio was used as the heart weight index. Left ventricular myocardial tissue and ileum tissue were fixed with 10% neutral formaldehyde. Using conventional methods, specimens were dehydrated and embedded in paraffin, and sectioning was followed by hematoxylin and eosin (H&E) staining and Masson staining, which were analyzed using optical microscopy. The images were generated by using an optical microscope (model BX-53F, Olympus, Japan).

### 16S rRNA gene sequencing analysis

Fresh feces were collected from rats in sterilized tubes, immediately frozen in liquid nitrogen upon collection, and stored in a refrigerator at −80°C until analysis. 16S rRNA gene sequencing analysis was completed by Beijing Nuohezhiyuan Technology Service Co., Ltd., China (Beijing, China), according to the manufacturer’s recommendations. Genomic DNA was extracted from the samples by CTAB (cetyltriethylammnonium bromide), and then the purity and concentration of DNA were measured by agarose gel electrophoresis. An appropriate amount of DNA was taken and diluted to 1 ng/μL in sterile water. According to the selection of the sequencing region, the diluted genomic DNA was taken as the template, the specific primer was used with Barcode, the Phusion of NEW England Biolabs^®^ High Fidelity PCR Master Mix was used with GC Buffer, and the efficient high-fidelity enzyme was used for PCR to ensure amplification efficiency and accuracy. The primers included the 16S V4 region primer (515F and 806R). PCR products were detected by electrophoresis on a 2% agarose gel. The qualified PCR products were purified by magnetic beads, quantified by enzyme labelling, and mixed in equal amounts according to the concentration of PCR products. After full mixing, PCR products were detected by 2% agarose gel electrophoresis, and the target strips were recovered using a Qiagen Gel Extraction Kit (Qiagen, Duesseldorf, Germany). TruSeq^®^ DNA PCR-Free Sample Preparation Kit (Illumina, San Diego, USA) was used for library construction. The concentration of the constructed library was quantified by Qubit and Q-PCR. After the library was qualified, NovaSeq6000 was used for machine sequencing.

After the barcode and primer sequences were removed from the sample data, raw tags were obtained by FLASH (V1.2.7, http://ccb.jhu.edu/software/FLASH/) splicing and then filtered to obtain high-quality clean tags. After tag quality control with reference to QIIME software (Version1.9.1, http://qiime.org/scripts/split_libraries_fastq.html), effective tags were finally obtained by removing the chimeric sequence. The Uparse algorithm (Version 7.0.1001, http://www.drive5.com/uparse/) was used to cluster all effective tags. By default, the sequences were clustered into OTUs (operational taxonomic units) with 97% identity. Additionally, representative sequences of OTUs were selected for species annotation. The mothur method and the SSUrRNA database of SILVA138 (http://www.arb-silva.de/) were used for species annotation analysis (the threshold was set as 0.8~1), and MUSCLE software (Version 3.8.31, http://www.drive5.com/muscle/) was used for rapid multiple sequence alignment to obtain the phylogenetic relationships of all OTUs representing sequences. Finally, the data obtained from each sample were homogenized.

Qiime software was used to calculate Shannon, Simpson, Chao1 and Ace indices. R software (Version 2.15.3) was used to draw rarefaction curves, rank abundance plots and species accumulation boxplots; analyze intergroup differences at the alpha-diversity level and beta-diversity level; and perform PCoA (Principal Co-ordinates Analysis) analysis (packages “stats” and”ggplot2”), NMDS (Non Metric Multi Dimensional Scaling) analysis (packages “vegan”), Anosim (Analysis of similarities) and MRPP (Multiple response permutation procedure) analysis (packages “vegan”,anosim function, mrpp function). LefSe (linear discriminant analysis effect size) analysis was performed using LefSe software, and the LDA (linear discriminant analysis) score was 4.

The Tax4Fun function was employed for the predictions of the functional profile of a microbial community based on 16S rRNA sequence data through the nearest neighbor method. The whole genome 16S rRNA gene sequence of prokaryotes in the KEGG (Kyoto Encyclopedia of Genes and Genomes) database was extracted and compared to the SILVA SSU Ref NR database (BLAST bitscore>1500) using the BLASTN algorithm. The OTUs of sequencing samples were clustered with the SILVA database sequence as a reference sequence, and then the functional annotation information was obtained.

### Statistical analysis

Statistical analyses were performed using IBM SPSS Statistics (Version 24.0, IBM, Armonk, NY, USA), and differences with a *P* value of <0.05 were considered to be statistically significant. All measurement data in the experiment were tested by the Shapiro−Wilk test for normal distribution. The measurement data with a normal distribution are expressed as the mean ± standard deviation for mean comparisons between multiple groups. One-way analysis of variance (ANOVA) was adopted, and the homogeneity of variance was first tested by Levene’s test. When homogeneity of variance was satisfied, the F test was used for population mean comparison, and the least-significant difference (LSD) test was used for pairwise comparison between groups. When homogeneity of variance was not met, the Welch test was used for population mean comparison, and the Games-Howell test was used for all statistical results for paired comparison between groups.

## Results

### General conditions, body weight, heart weight, heart/body weight ratio and survival rate

During weeks 1-3 of the experiment, the rats in each group usually drank and had soft hair and free activity, and their body weight showed an increasing trend. During weeks 4-6, drug poisoning effects were observed in the DOX group, mainly manifested as a poor mental state, decreased activity, increased eyelid secretion, decreased intake of food and water, depilation, and accelerated respiration. Some rats showed ocular congestion, diarrhea and other symptoms. The body weight of the DOX-A to DOX-F groups decreased to varying degrees, but the body weight of the DOX-G to DOX-I groups still increased slightly, and no abnormal manifestations were observed in the CON-J and CON-K groups. At the end of the sixth week, eight rats survived in the DOX-B group, seven in the DOX-F and DOX-I groups, and nine in other groups.

**Figure 2:**
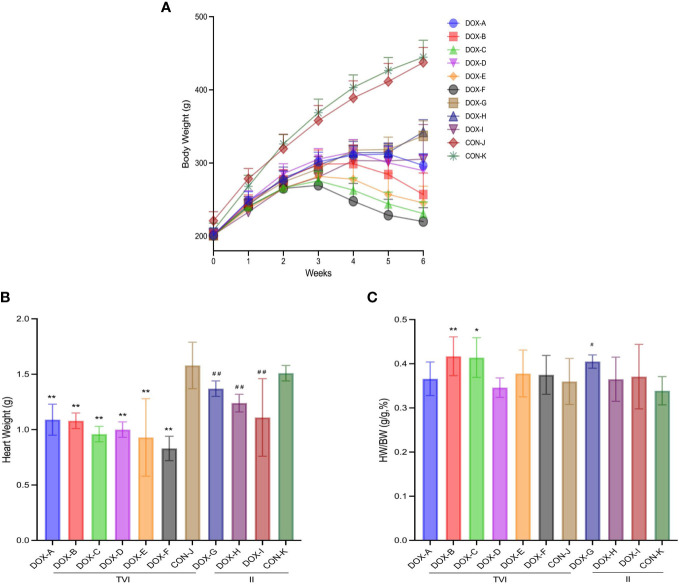
Weight results of rats in each group. **(A)** Change of body weight (BW) during modeling period. **(B)** Heart weight (HW). **(C)** BW/HW ratio. CON, control; DOX, doxorubicin; TVI,tail vein injection; II,intraperitoneal injection. Data are presented as means ± SD (n = 7,8 or 9 per group). ^*^ *P* < 0.05, ^**^ *P* < 0.01 vs. CON-J; ^#^ *P* < 0.05, ^##^ *P <*0.01 vs. CON-K.

After the cardiac function examination finished at the end of the experiment, the abdominal cavity of the rats was opened. Some rats in the DOX-C and DOX-F groups showed hepatic oedema and occasionally slight ascites; slight hepatic oedema with unequal pleural effusion and a small amount of ascites were occasionally observed in the DOX-G group, hepatic oedema with unequal pleural effusion and ascites were commonly observed in the DOX-I group, while other groups showed no apparent abnormalities. Consistent with previous reports (Miao et al, 2015; You, 2015; Zhang et al, 2018). The body weight, heart weight, and heart/body weight ratio are shown in **Figures 2A–C**.

### Myocardial injury induced by doxorubicin

**Figure 3:**
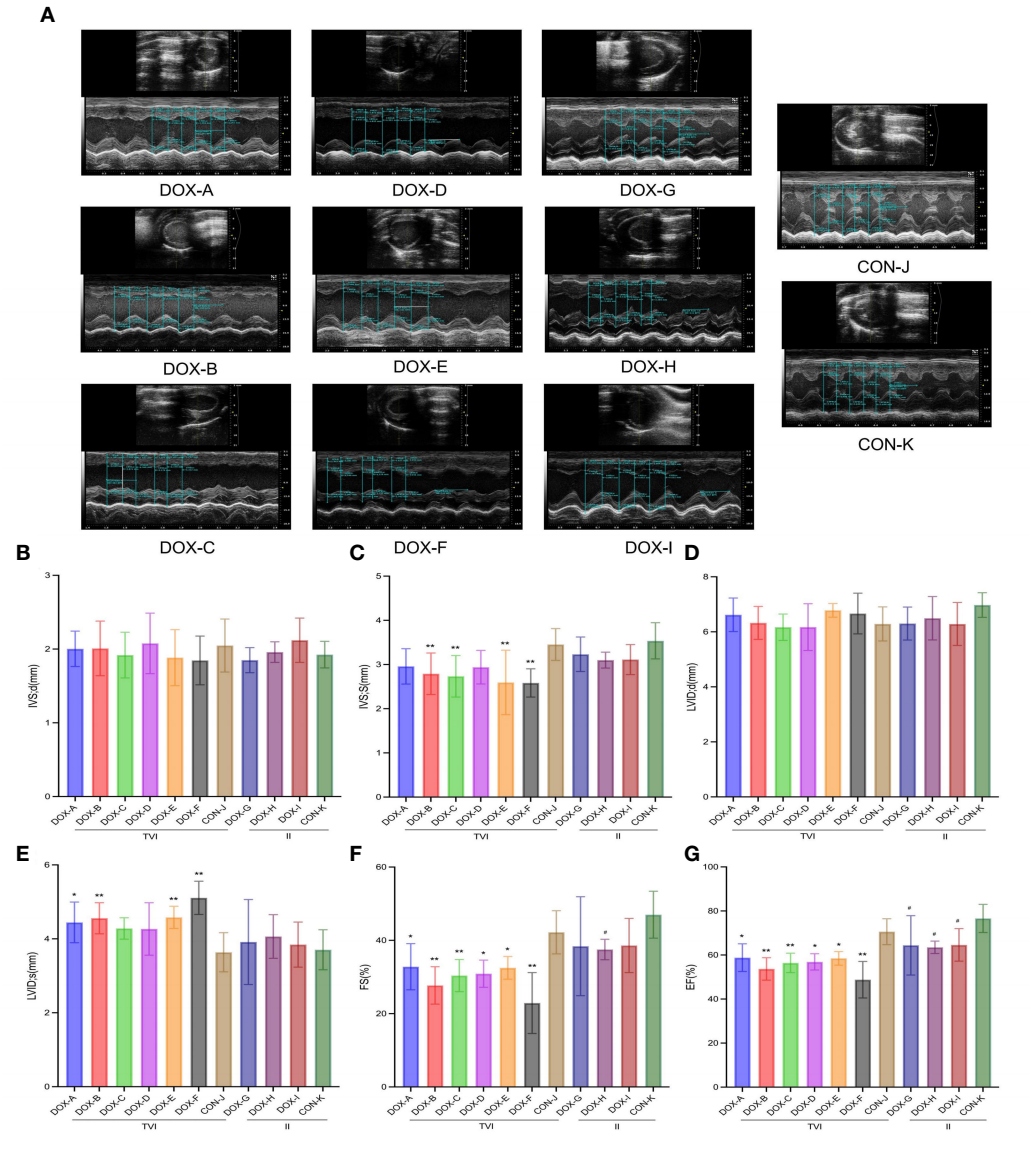
DOX-induced cardiac dysfunction and histological injury. **(A)** Representative echocardiograms. **(B–G)** Comparison of echocardiography parameters. LVID;d, left ventricular internal diameter at end-diastole; LVID;s, left ventricular internal diameter at end-systole; IVS;d, interventricular septal thickness at end-diastole; IVS;s, interventricular septal thickness at end-systole; FS%, left ventricular fractional shortening; EF%, left ventricular ejection fraction. CON, control; DOX, doxorubicin. *P < 0.05, **P < 0.01 vs. CON-J; #P < 0.05, ##P <0.01 vs. CON-K.

Representative two-dimensional echocardiography and comparisons of various parameters after 6 weeks of intervention in all groups are shown in **Figures 3A–G**. The ventricular septum in the DOX-B, C, E and F groups was thinner to varying degrees than in the CON-J group, the left ventricular dilatation in the DOX-A, B, E and F groups was different to different degrees, and the left ventricular emptying ability and left ventricular contractility in the DOX-A to DOX-F groups were decreased to varying degrees, which was the most obvious in the DOX-F group, with a statistically significant difference (*P* < 0.05). The left ventricular emptying ability in the DOX-H group was lower than that in the CON-K group, and the left ventricular contractility of the DOX-G to DOX-I groups was decreased to varying degrees; the difference was the most obvious in the DOX-H group, with a statistically significant difference (*P* < 0.05).

**Figure 4:**
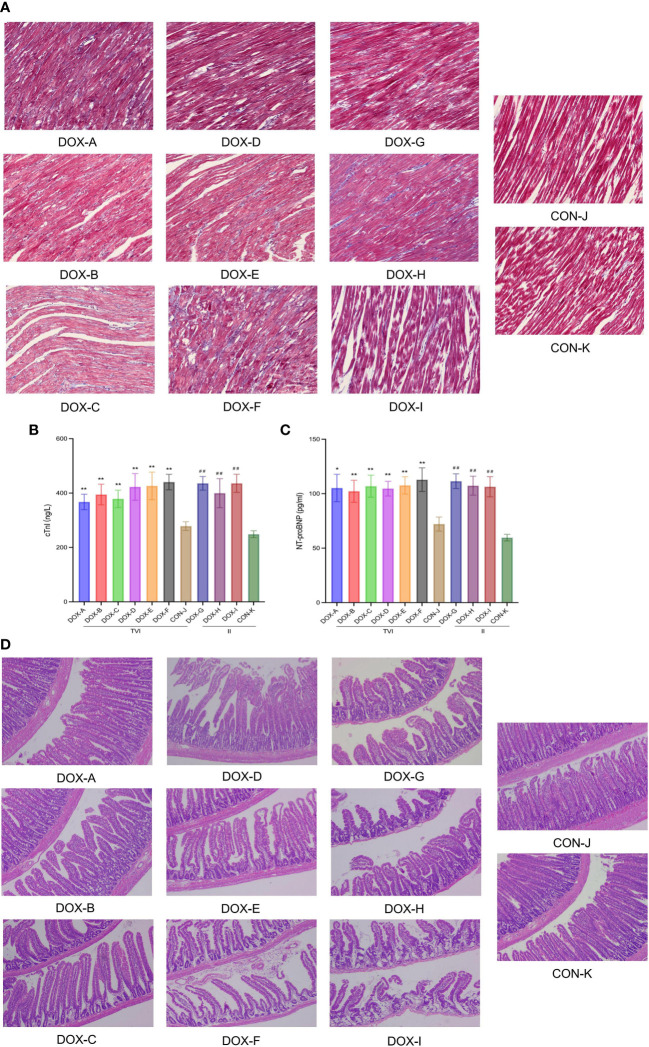
**(A)** Myocardial fifibrosis observed under a light microscope with Masson staining (Magnification ×200, Scale bar = 100 µm). **(B, C)** The levels of cTnI and NT-proBNP in the serum.CON, control; DOX, doxorubicin; TVI,tail vein injection; II,intraperitoneal injection. Data are presented as means ± SD (n = 5).**(D)** Pathological changes observed under a light microscope with H&E staining (Magnification ×200, Scale bar = 100 µm). CON, control; DOX, doxorubicin.^*^ *P* < 0.05, ^**^ *P* < 0.01 vs. CON-J; ^##^ *P <*0.01 vs. CON-K.

Myocardial collagen deposition was detected by Masson’s trichrome staining (**Figure 4A**). Unlike in the CON groups, there was no apparent myocardial fibrosis in the DOX-A, D, and G groups; focal fibrotic hyperplasia was observed in the DOX-B, E, H, and I groups; and diffuse fibrosis was observed in the DOX-C and F groups, with the most severe myocardial fibrosis in the DOX-F group. NT-proBNP reflects the level of damage to ventricular pressure and cardiac function, and cTnI is a marker of myocardial injury. Both are sensitive and reliable diagnostic biomarkers of heart failure. The serum cTnI and NT-proBNP levels in the DOX-A to DOX-F groups were higher to varying degrees than those in the CON-J group, and the most apparent increase was in the DOX-F group; the difference was statistically significant (*P* < 0.05). The serum cTnI and NT-proBNP levels in the DOX-G to DOX-I groups were higher to varying degrees than those in the CON-K group, the serum cTnI level was increased most obviously in the DOX-I group, and the serum NT-proBNP level was increased most obviously in the DOX-G group, and the difference was statistically significant (*P* < 0.05) (**Figures 4B, C**). The above results suggested that the severity of myocardial injury induced by DOX differs depending on different doses and modes of administration.

### Intestinal mucosal injury induced by doxorubicin

Histopathological sections of intestinal tissues were observed under a light microscope (**Figure 4D**). In the CON groups, the intestinal tissue structure was intact, the mucosa was arranged neatly, the glands were not atrophic, and there was no apparent inflammatory cell infiltration under the mucosa. In the DOX-A to DOX-G groups, the intestinal tissue structure was relatively complete, with partial loss of intestinal mucosal integrity, oedema of the colon mucosal epithelium, atrophy of a few glands, and partial inflammatory cell infiltration in the mucosal lamina propria. In the DOX-A to DOX-G groups, the intestinal tissue structure was incomplete, with complete loss of intestinal mucosal integrity, atrophy of most glands, thinning of mucosal lamina propria and separation of villi from the basement membrane. The pathological changes in intestinal tissue in the DOX-I group were the most serious.

### Diversity analysis of gut microbiota

To characterize the effect of DOX on gut microbial communities, a PCR-free library was constructed based on the Illumina Nova sequencing platform, and paired-end sequencing was performed. By splicing reads and quality control, the effective rate of quality control reached 76.48%. The sequences were clustered into OTUs with 97% identity. Then, the OTU sequences were annotated with the Silva138 database. These reads were matched into 3049 OTUs, including 37 phyla, 70 classes, 209 families, 382 genera, and 241 species of gut microbes. Petal diagrams and Venn diagrams were used to visualize and compare commonalities and characteristics of species (such as OTUs) from each group of samples.

**Figure 5:**
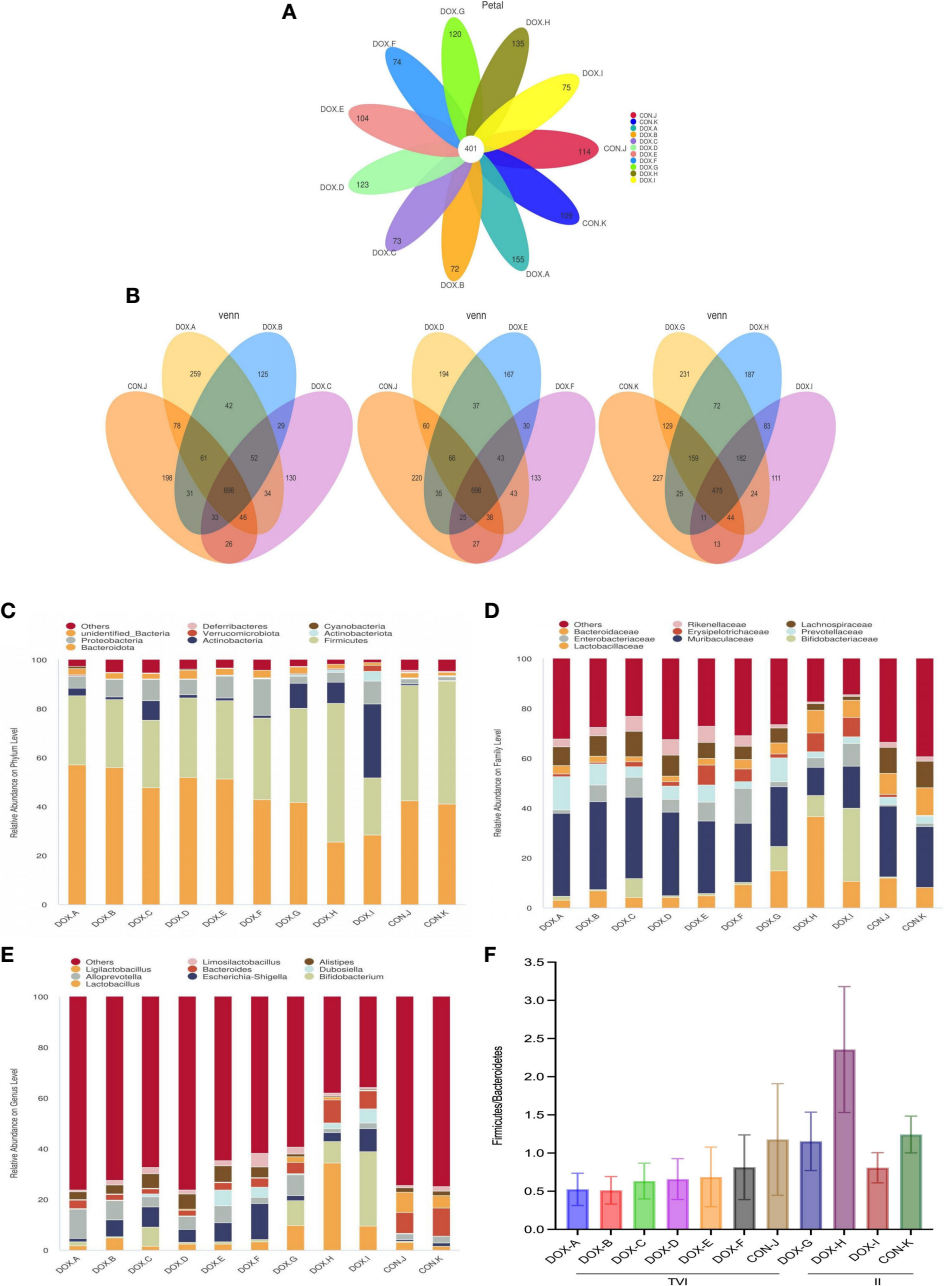
OTU analysis and compositionof the gut microbiota. **(A)** Petal diagram. **(B)** Venn diagram.*Each circle in the Petal or Venn diagram represent a group of samples. The number of circles and overlapped parts represents the number of OTUs shared between the sample groups, and the number without overlapped parts represents the number of OTUs unique to the sample group. **(C–E)** Relative abundance of microbial community from each group of rat at phylum, family, and genus levels. **(F)**The ratio of Firmicutes: Bacteroidetes (F/B) at the phylum level.CON,control; DOX, doxorubicin. n = 4.

As shown in the Petal diagram, the total numbers of unique OTUs in each group were 155 (DOX-A), 72 (DOX-B), 73 (DOX-C), 123 (DOX-D), 104 (DOX-E), 74 (DOX-F), 120 (DOX-G), 135 (DOX-H), 75 (DOX-I), 114 (CON-J), and 109 (CON-K) (**Figure 5A**), respectively. As shown in the Venn diagrams, the total numbers of unique OTUs were 259 (DOX-A), 125 (DOX-B), 130 (DOX-C), differed from 196 (CON-J); the total numbers of unique OTUs were 231(DOX-G), 187(DOX-H), 111(DOX-I), differed from 227(CON-K) (**Figure 5B**). The results showed that the number of OTUs in the DOX groups were lower to different degrees than those in the CON groups.

Based on the species annotation results, the top 9 microbiota with high relative abundance at the phylum, family, and genus levels were selected to generate column accumulation diagrams (**Figures 5C–E**). The ratio of Firmicutes : Bacteroidetes (F/B) in all groups was observed at the phylum level (**Figure 5F**). The results showed that the GM compositions in the DOX and CON groups were different. At the phylum level, the top 3 dominant species in the DOX-A to DOX-F groups were Bacteroidetes, Firmicutes and Proteobacteria; in the DOX-G group, they were Bacteroidetes, Firmicutes and Actinobacteria; in the DOX-H group, they were Firmicutes, Bacteroidetes and Actinobacteria; in the DOX-I group, they were Actinobacteria, Bacteroidetes and Firmicutes; in the CON-J and CON-K groups, they were Firmicutes, Bacteroidetes and Proteobacteria. At the family level, the most dominant species in each group was Muribaculaceae. The GM in the DOX-A to DOX-F groups featured a lower percentage of Firmicutes and a higher percentage of Bacteroidetes than the GM in the CON-J group, and the ratio of Firmicutes : Bacteroidetes (F/B) at the phylum level was lower to varying degrees. The GM in the DOX-G group featured a lower percentage of Firmicutes and a higher percentage of Bacteroidetes than the GM in the CON-K group, the DOX-H group featured a higher percentage of Firmicutes and a lower percentage of Bacteroidetes, the DOX-I group featured a lower percentage of Firmicutes and Bacteroidetes; the ratio of Firmicutes : Bacteroidetes (F/B) at the phylum level in the DOX-G and DOX-I groups were decreased to varying degrees, and the trend in the DOX-H group was the opposite.

### Sample complexity analysis

Alpha diversity was used to analyze the microbial community diversity. The rarefaction curve can directly reflect the rationality of the amount of sequencing data, and the results showed that the curves eventually tended to plateau, indicating that the sequencing depth was sufficient and that the data were reasonable (**Supplementary Figure S1A**).The rank abundance plot can reflect the richness and evenness of species in the sample. In the horizontal direction, the span of the curves on the horizontal axis was significant, indicating that each group’s community abundance of GM was high. In the vertical direction, the curves tended to be consistent, indicating that each group’s community distribution of GM was relatively uniform (**Supplementary Figure S1B**). The results of the species accumulation boxplot showed that the box plots tended to be gentle, indicating that the sample size of the sequencing was sufficient, and the amount of existing sequencing data could reflect the diversity information of bacterial flora in the sample (**Supplementary Figure S1C**).

**Figure 6:**
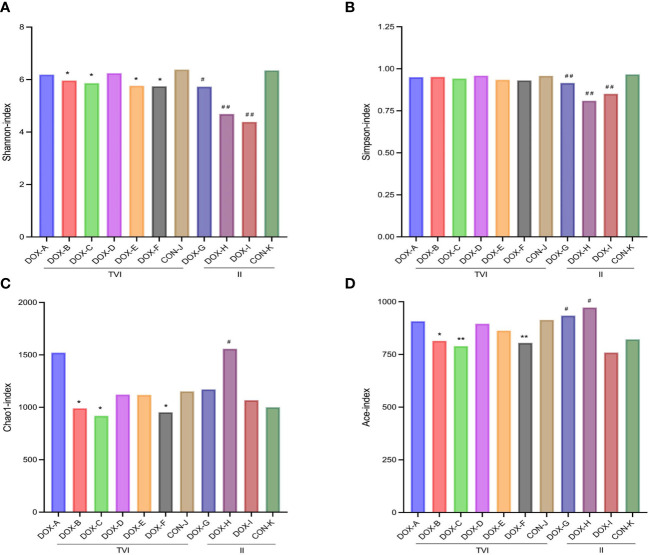
Alpha Diversity. **(A–D)** Shannon,Simpson,Chao1and ACE index. CON, control; DOX, doxorubicin; OTU, operational taxonomic unit; TVI,tail vein injection; II, intraperitoneal injection. ^*^ *P* < 0.05, ^**^ *P* < 0.01 vs. CON-J; ^#^ *P* < 0.05, ^##^ *P* < 0.01 vs. CON-K.

The Shannon and Simpson indices based on OTU distribution can represent the diversity of GM in the sample. The Chao and ACE indices can represent the richness of GM in the sample. The changes in Shannon, Simpson, Chao1 and ACE indices for each group are shown in the bar plot (**Figures 6A–D**). The results showed that the Shannon index in the DOX-B, C, E, F groups and the Chao1 index and ACE index in the DOX-B, C, F groups were lower than those in the CON-J group (*P* < 0.05, *P* < 0.01); the Shannon index and Simpson index in the DOX-G, H, I groups were lower than those in the CON-K group (*P* < 0.05, *P* < 0.01); the Chao index and ACE index in the DOX-H group and the ACE index in the DOX-I group were higher than those in the CON-K group (*P* < 0.05). The above results showed that the microbial diversity and richness in the DOX-B, C, F groups were lower than those in the CON-J group; the microbial diversity in the DOX-G and DOX-H groups were lower, but the microbial richness was higher than in the CON-K group.

ANOSIM MRPP can be performed by directly utilizing the OTU relative abundance table to analyze whether the differences in microbial community structure between groups are significant and whether the differences between groups are greater than the differences within groups to judge whether the grouping is meaningful. The results showed differences between DOX groups under different schemes, the differences between groups were significantly greater than the differences within groups, and the correlation between groups’ differences was substantial and statistically significant (**Supplementary Table S1**).

**Figure 7:**
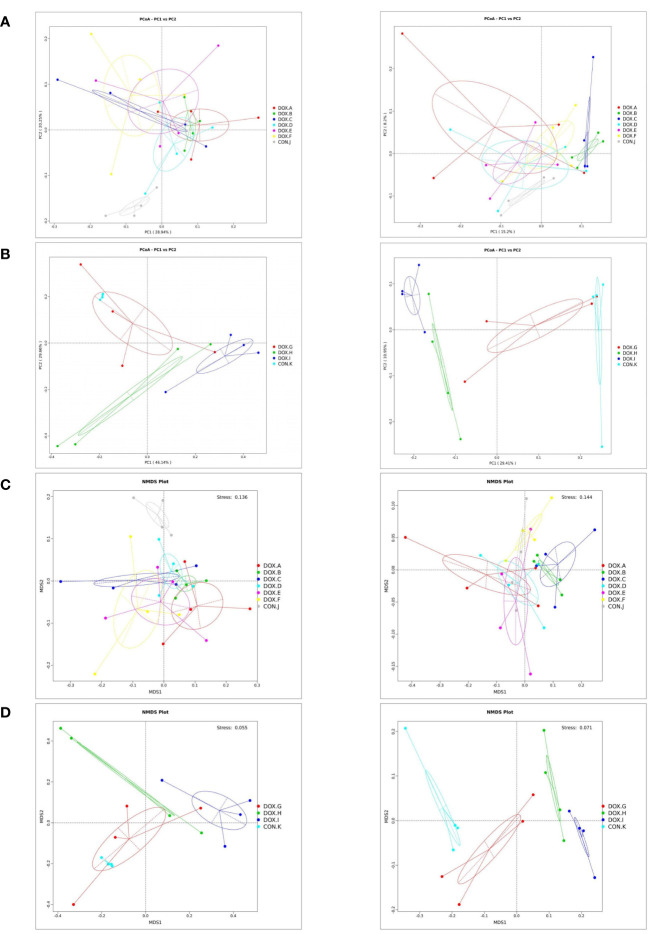
Cluster analysis by PCoA and NMDS analysis of the gut microbiota. **(A, B)** PCoA of weighted and unweighted unifrac distances. PCoA1 and PCoA2 represented the contribution of principal components to sample differences.*The more similar the species composition structure, the closer the samples were to each other. Therefore, the samples with high similarity in community structure tended to cluster together, while the samples with very different communities were far apart. **(C, D)** NMDS of weighted and unweighted unifrac distances. Species information contained in samples were reflected in the multi-dimensional space in the form of points, and the distance between points reflected the degree of difference between different samples, which can reflected the inter-group and intra-group differences of samples. Stress < 0.2 can accurately reflect the degree of difference between samples. CON, control; DOX, doxorubicin; PCoA, Principal Co-ordinates Analysis; NMDS,Non Metric Multi Dimensional Scaling.

PCoA and NMDS can accurately observe the degree of difference between samples and the variation rules of the differences. The results showed that the samples in each group were clustered, indicating that the similarity of samples in the group was relatively high. The dispersion of samples between groups was observed, and the separation of samples between the DOX and CON groups was pronounced (**Figure 7**). Therefore, the DOX groups possessed distinct differences in the unique aggregation of faecal microbial structures from the CON groups.

**Figure 8:**
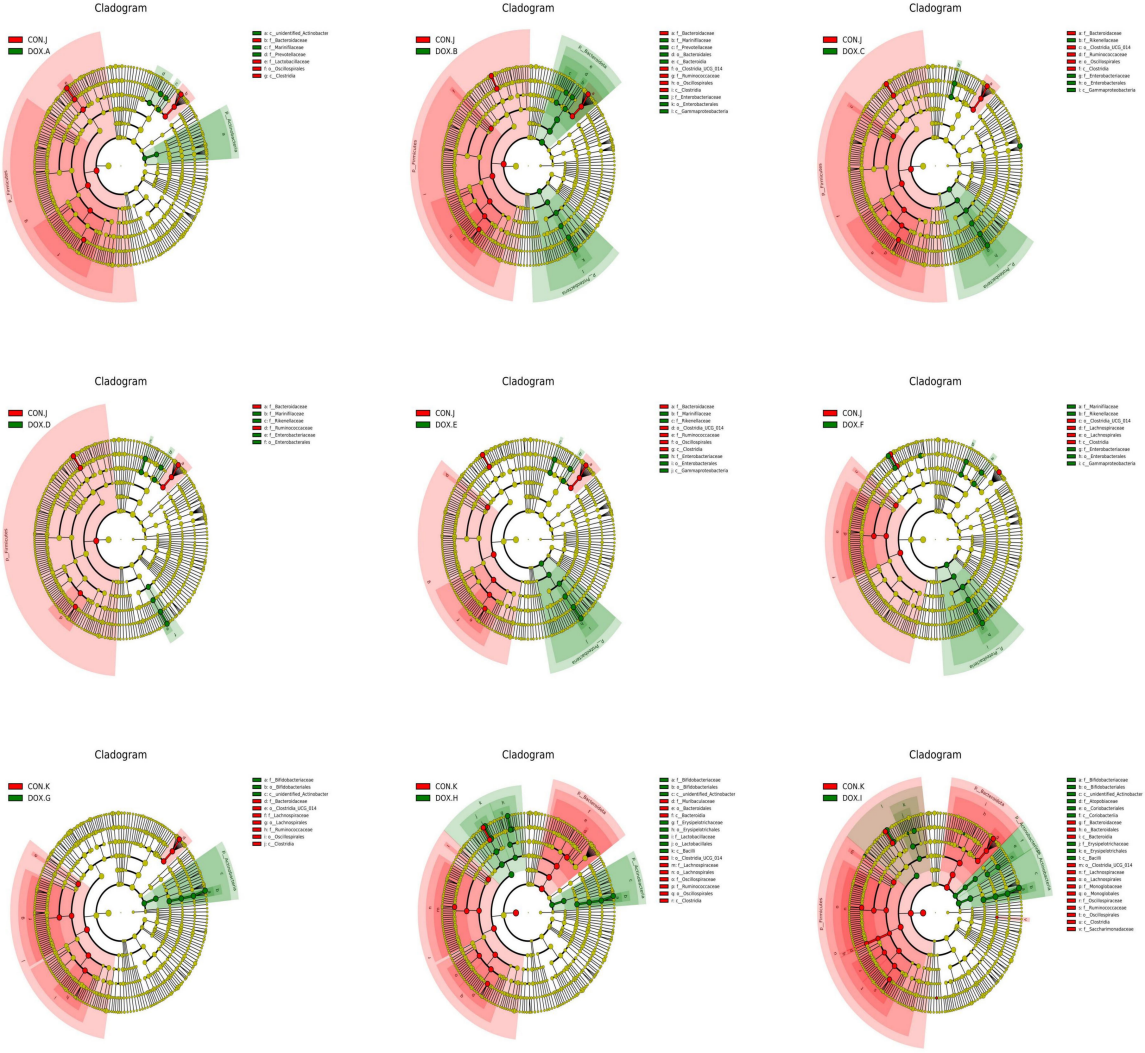
Histogram analyzed by LEfSe (LDA = 4.0, *P* < 0.05) showing the LDA scores for differentially abundant genera in CON group and DOX group. Circles radiating from inside to outside indicate phylogenetic levels from kingdom to genus. Nodes with different colors indicate microbial taxa that are enriched in the corresponding groups and have significant differences between groups, and the size of node diameter is proportional to the relative abundance size; yellow nodes indicate microbial taxa that have no significant differences between groups. CON, control; DOX, doxorubicin; LDA, linear discriminant analysis; LEfSe, linear discriminant analysis effect size. n = 4.

**Figure 9:**
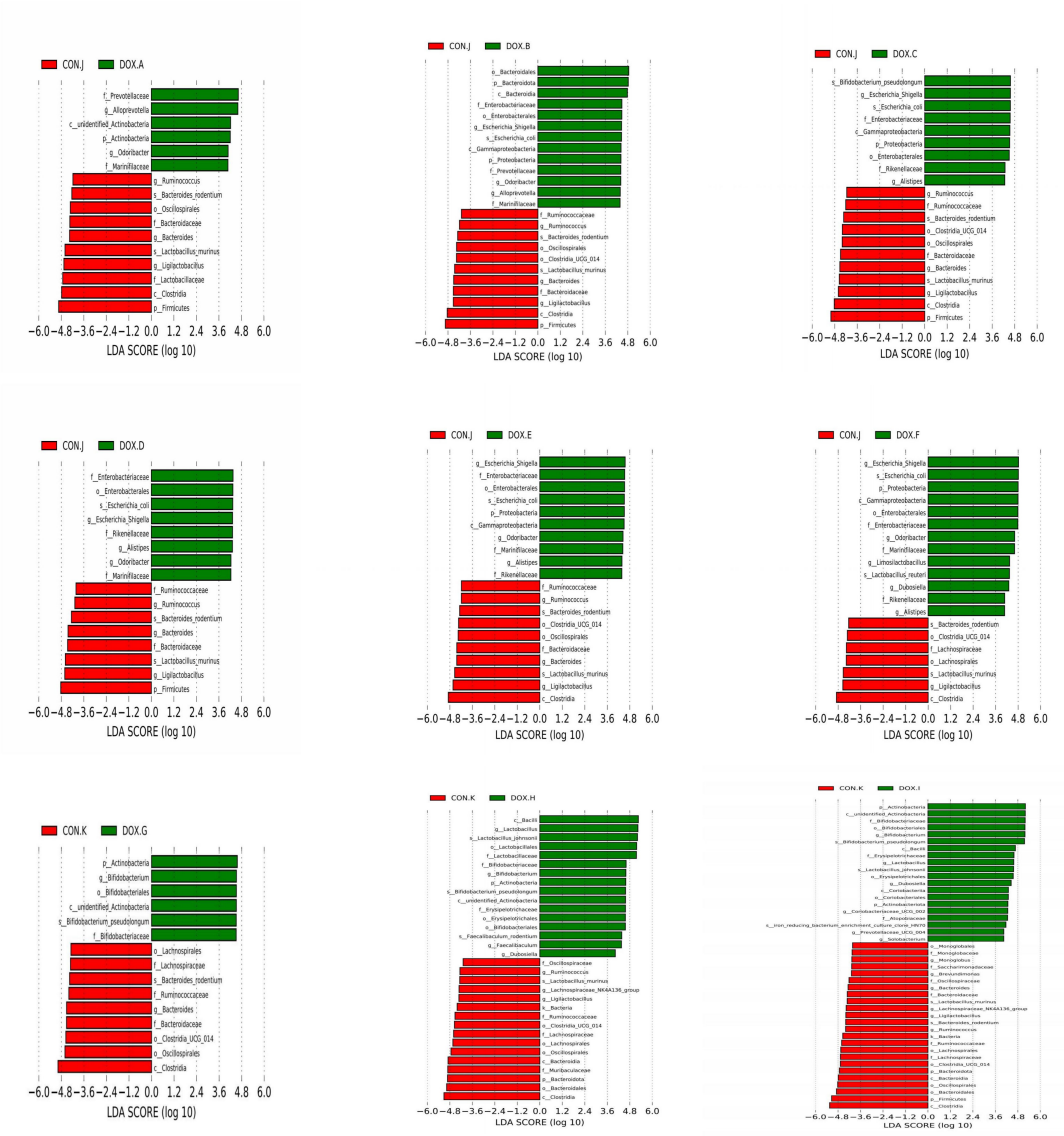
Cladogram analyzed by LEfSe (LDA = 4.0, *P* < 0.05) showing the phylogenetic distribution of the bacterial lineages in CON group and DOX group. CON, control; DOX, doxorubicin; LDA, linear discriminant analysis; LEfSe, linear discriminant analysis effect size. n = 4.

LEfSe analysis based on discriminative features was performed to identify specific bacterial taxa with significant differences between the CON and DOX groups. The threshold of the LDA score was set to 4.0, and species exceeding the set value were considered biomarkers with significant differences between groups; phylogenetic branching maps were drawn according to the different species. This analysis identified 5 phyla and 18 families, and the main nodes in the DOX groups were concentrated in p_Actinobacteria(DOX-A,G,H,I), p_Proteobacteria(DOX-B,C,E,F), p_Bacteroidota(DOX-B), p_Actinobacteriota (DOX-I), f_Marinifilaceae (DOX-A, B, D, E, F), f_Prevotellaceae (DOX-A, B), f_Enterobacteriaceae (DOX-B,C,D,E,F), f_Rikenellaceae(DOX-C,D,E,F), f_Bifidobacteriaceae(DOX-G,H,I), f_Erysipelotrichaceae(DOX-H,I),f_Lactobacillaceae (DOX-H) and f_Atopobiaceae (DOX-I); the main nodes in the CON groups were concentrated in p_Firmicutes (CON-J,K), f_Bacteroidaceae (CON-J, K), f_Lactobacillaceae (CON-J), f_Ruminococcaceae (CON-J, K), f_Lachnospiraceae (CON-J, K), f_Muribaculaceae (CON-K), f_Oscillospiraceae (CON-K), f_Monoglobaceae (CON-K) and f_Saccharimonadaceae (CON-K), which were responsible for this discrimination (**Figure 8**). In the DOX groups, the relative abundances of p_Bacteroidetes (DOX-B), p_Proteobacteria (DOX-B,C,F), p_Actinobacteria (DOX-G,H,I), f_Prevotellaceae (DOX-A,B), f_Enterobacteriaceae (DOX-B), f_Lactobacillaceae (DOX-H), f_Enterobacteriaceae (DOX-C,D,E,F), f_Marinifilaceae (DOX-E,F), f_Bifidobacteriaceae (DOX-G,H,I), f_Erysipelotrichales (DOX-H,I), g_Alloprevotella (DOX-A,B), g_Escherichia_Shigella(DOX-B,C,D,E,F),g_Odoribacter(DOX-B), g_Odoribacter(DOX-D,E,F),g_Bifidobacterium (DOX-G,H,I), g_Lactobacillus (DOX-H,I), g_Faecalibaculum(DOX-H) and g_Dubosiella (DOX-I) were significantly higher and the abundances of p_Firmicutes (CON -J,K), p_Bacteroidota (CON-K), f_Bacteroidaceae (CON-J,K), f_Lactobacillaceae (CON-J), f_Ruminococcaceae (CON-K), f_Muribaculaceae (CON-K), f_Lachnospiraceae (CON-K), g_Bacteroides (CON-J, K) and g_Ligilactobacillus (CON-J) were significantly lower than those in the COX groups (**Figure 9**).

### Tax4Fun function prediction

**Figure 10:**
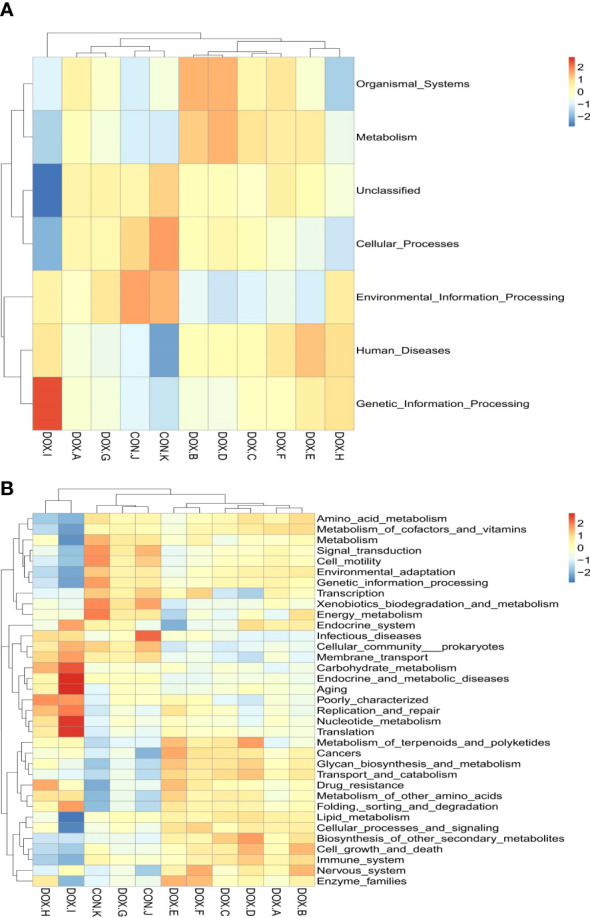
Tax4Fun analysis of the gut microbiota. **(A, B)** Feature annotation clustering heatmap at Tax4Fun level 1 and level 2. CON, control; DOX, doxorubicin.

**Figure 11:**
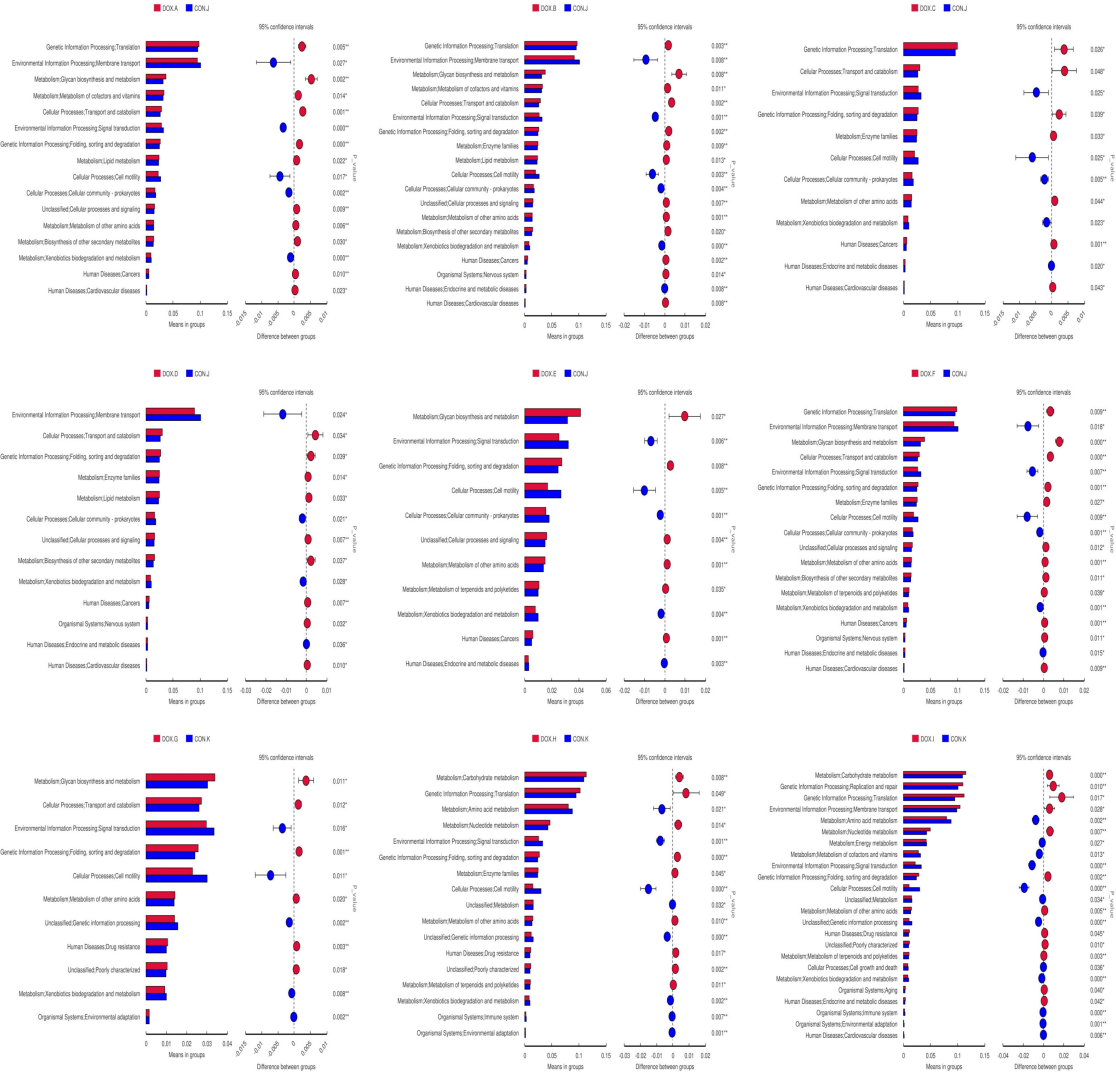
Predicated function of the gut microbiota based on Tax4Fun level 2 Differential metabolic pathway in CON group and DOX group. CON, control; DOX, doxorubicin.

Tax4Fun was used to predict the metabolic function of intestinal microorganisms in the CON and DOX groups, and a total of 7 KEGG primary metabolic pathways were annotated (**Figure 10A**), including cellular processes, organic system, metabolism, cellular processes, environmental information, human disease, genetic information processing and Unclassified. Thirty-five KEGG secondary metabolic pathways were further annotated (**Figure 10B**). A t test was used for each type of metabolic pathway to calculate the intergroup differences between the CON and DOX groups, and the results are shown in **Figure 11** (*P* < 0.05) and (**Supplementary Table S2**). The above results showed that the DOX and CON groups had multiple functional pathways in the GM that were completely different.

## Discussion

As HF is a global healthcare epidemic, it is vital to establish a well-established and stable animal model to explore potential pathogenic mechanisms and identify new therapeutic targets for HF. The induction of congestive HF by DOX has been used for a long time, and this approach to establish animal models has also been recognized. The myocardial toxicity model of DOX can be found during the early stage of the experiment after DOX administration (Huang et al, 2022). Since the incidence and severity of heart failure are directly related to the accumulation of DOX in the body and have a cumulative effect over time, the condition can evolve into HF during the later stage of the experiment with further aggravation of the injury. There have been many studies on the model of DOX-induced HF in rats conducted globally, but the route, mode and total cumulative dosage of administration are different (Cheng et al., 2011; Wu et al., 2014; Zhou et al., 2014). Here, for the first time, rat models of HF under different administration schemes were used, which include two routes of administration (TVI and II), two modes of administration (fixed dose and alternating dose), and three total cumulative doses (12 mg/kg, 15 mg/kg and 18 mg/kg). The results showed that the DOX groups showed different degrees of myocardial contractility reduction, myocardial fibre arrangement disorder and myocardial injury than the CON groups. The condition in the DOX-F group was the most serious, with statistically significant differences. This study indicates that the degree of HF induced in the model is the most severe and stable when TVI gives the rats at doses of 4 mg/kg and 2 mg/kg that is alternately administered once a week for 6 consecutive weeks and a total cumulative dose that reaches 18 mg/kg, this approach can be used as an ideal method to establish an animal model of HF. Here, the causes of this result are analyzed.

The route of administration is divided into II and TVI. First, after II, DOX needs to be absorbed through the peritoneum before entering the blood circulation and finally reaching all body organs, whereas DOX administered through TVI can directly enter the blood and act on the heart, which can maximize the cardiotoxicity of DOX. Second, with the accumulation of the DOX dose during the later period following II, rats are prone to ascites, greater omentum swelling, liver oedema and other reactions (Kobayashi et al., 2004; Wu et al., 2012); the existence of ascites may lead to a misjudgment of the weight of the rats, and the dose administered is proportional to body weight; thus, a higher dose of DOX may be administered, which creates a vicious cycle of increased DOX dosing, drug toxicity, ascites formation, and weight gain, which leads to a further increase in the dose of DOX. Additionally, ascites may affect the absorption of DOX by the heart, which prevents the predetermined blood concentration from reaching; however, such phenomena are less likely to occur with TVI. Finally, II easily causes local necrosis of the abdominal skin in rats, and a large number of drugs directly stimulate the abdominal cavity locally and cause more obvious gastrointestinal toxicity, increased risk of death in rats, increased difficulty in observation and intervention during the later stages of the experiment, and poor reproducibility of the model; however, using TVI avoids the direct stimulation of the gastrointestinal tract.

The mode of administration is divided into fixed dose administration and alternating dose administration. The effects of the mode of administration may have resulted from the fact that alternating doses were administered at high doses during weeks 1, 3 and 5 of the modelling period to cause myocardial injury rapidly, and low doses were applied during weeks 2, 4 and 6 of the modelling period to ensure a continuous state of myocardial injury; thus, fixed dose was more likely to cause drug tolerance, so alternating doses could more effectively induce the cardiotoxicity associated with DOX, and the degree of HF was more pronounced.

The total cumulative dose of administration was divided into three gradients, 12 mg/kg, 15 mg/kg and 18 mg/kg, and literature reports have shown that when DOX is administered, a single dose is generally maintained at 1-6 mg/kg, the total cumulative dose is usually controlled at 12-20 mg/kg (Xu et al., 2016; Yu and Wang, 2018), doses below 12 mg/kg are usually less likely to trigger HF(Ma, 2019), and successful modelling can be achieved up to a dose of 15 mg/kg (Hayward and Hydock, 2007). However, when the dose is too high, other toxic side effects may occur, leading to death. The experimental results showed that the dose of 18 mg/kg was the best, consistent with literature reports.

**Figure 12:**
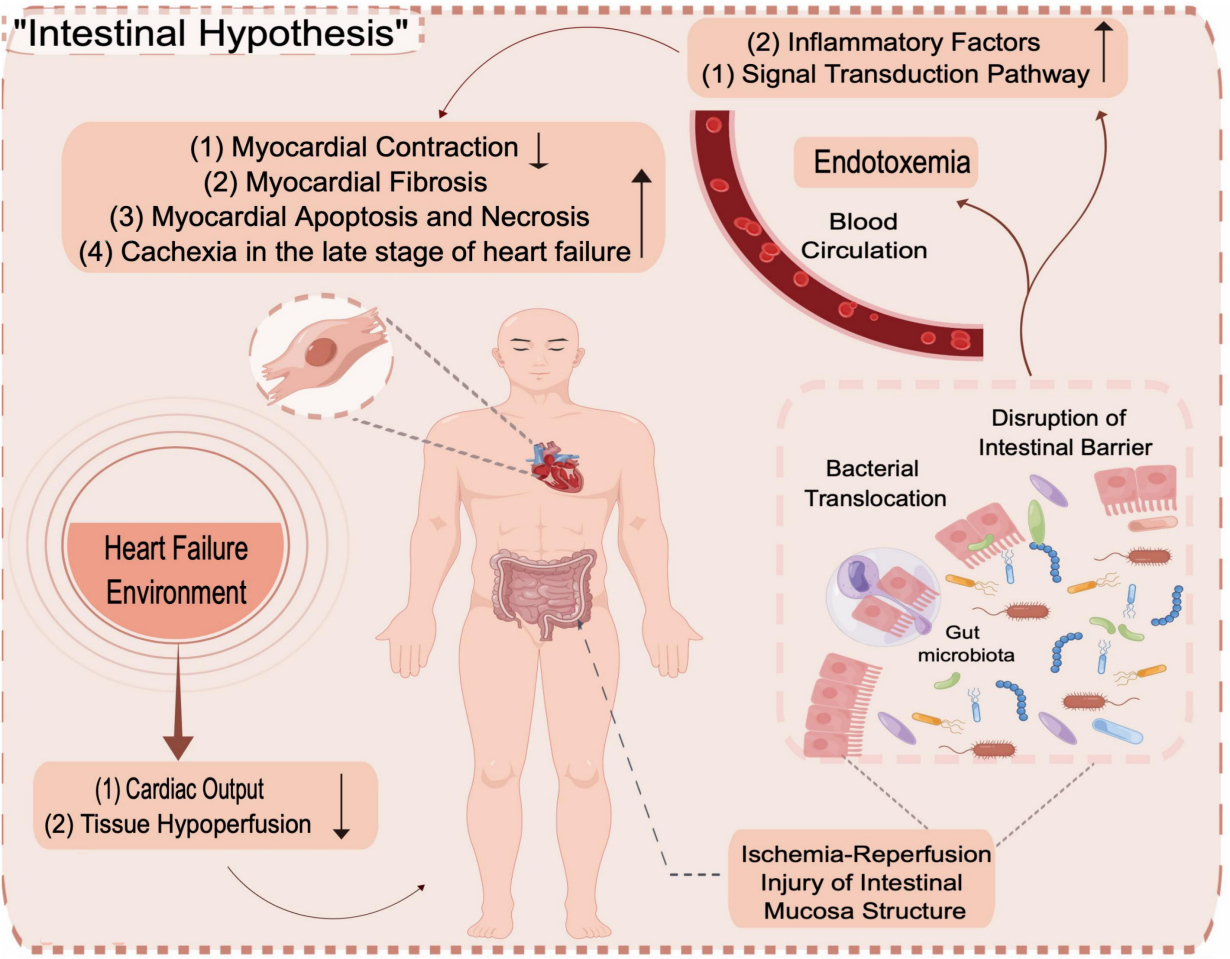
Intestinal Hypothesis.

In the past two decades, the GM has been a target for studying the pathogenesis of congestive HF and drug intervention (Drapkina et al., 2022). To date, studies on the correlation between HF and the GM mostly begin with studies of circulatory impairment, insufficient arterial filling and systemic organ stasis caused by HF, which manifests in the gut as intestinal stasis, hypoperfusion, barrier dysfunction, increased permeability, increased bacterial biofilm production and dysregulation of the GM (Nagatomo and Tang, 2015; Liu and Liu, 2022), resulting in systemic inflammatory hyperresponsiveness due to GM microbe translocation. The dysregulated microbiota further affects the pathophysiological process of HF by mediating an inflammatory reaction and inducing adverse reactions such as cachexia during the late stages of HF (Guo et al., 2018; Song and Zhou, 2019), aggravating the clinical symptoms of HF and causing adverse effects on prognosis (**Figure 12**). Recently, most studies on HF and the GM have been based on intrapopulation correlation studies, in which individual differences and the evolution of the disease in HF patients have been found to impact the study’s performance and the generation of results. The need for researchers to simultaneously analyze the structural changes in the GM caused by HF and the inability to define the study variables make such studies very difficult clearly.

The dynamic balance formed by the interaction of the GM with the intestinal mucosal barrier, nutrients and metabolites is called intestinal homeostasis.THE GM is crucial for maintaining intestinal homeostasis (Ren et al., 2019). Altered GM and impaired gut barrier function in the context of HF disrupt intestinal homeostasis, leading to abnormal production and absorption of microbial-derived metabolites, the expansion of potentially pathogenic microorganisms and the depletion of microorganisms with anti-inflammatory properties, which are features of the composition of the GM in the context of HF; the translocation of bacterial metabolites (e.g., LPS, TMAO) enhances interactions with the gut and systemic innate immune system, exacerbating cardiac insufficiency, systemic inflammation and malnutrition in patients with HF (Kamo et al., 2017b; Tang et al., 2019; Drapkina et al., 2022). In this study, the effect of DOX on the structure of the GM in rats with HF under different administration schemes was investigated by intestinal HE staining combined with 16S rRNA high-throughput sequencing technology. Sequencing can not only be used to identify the distribution of the abundance of flora at different taxonomic levels and explore the differences in community structure between different groups but also to evaluate microbial communities for functional prediction.

The experimental results showed that the GM’s structural composition and functional network significantly different between the CON and DOX groups of rats with intestinal damage. The number of specific OTUs in the DOX-C, F and I groups was lower than in the CON groups. According to alpha diversity analysis, the Shannon, Simpson, and Chao1 indices in the DOX-B, C and F groups and the Shannon and Simpson indices in the DOX-G, H and I groups were all lower than those in the CON groups, with statistically significant differences. The study showed that DOX significantly reduced the species diversity of rat faecal bacteria, consistent with other studies that suggested that DOX significantly reduced the species diversity of rat faeces (Wu et al., 2019). It was speculated that the reason underlying this change might be that the relative abundance of harmful microbiota increased and the propagation of normal microbes was inhibited, thus reducing the diversity of the GM.

In the human intestinal microecosystem, Firmicutes, Bacteroidetes, Actinobacteria and Proteobacteria are the four most dominant groups in the intestine and are similar to those in SD rats at the phylum level (Ley et al., 2006; Human Microbiome Project Consortium, 2012). Studies have shown that most of the Firmicutes bacteria are butyric acid-producing. Butyric acid, as one of the final metabolites of polysaccharides that are not absorbed by the fermenting intestinal bacteria within the host, can provide energy to the host and promote the development of intestinal epithelial cells (Liu et al., 2012), reduce the expression of proinflammatory cytokines, attenuate the intestinal inflammatory response (Segain et al., 2000) and protect the integrity of the intestinal epithelial barrier by inhibiting the activation of nuclear factor (NF)-κB and stimulating the production of mucin and antimicrobial peptides; additionally (Segain et al., 2000; Canani et al., 2011), a reduction in the abundance of Firmicutes can induce or exacerbate the local inflammatory response. A decrease in the ratio of Firmicutes to Bacteroidetes (F/B ratio) represents a disruption of homeostasis in the gut and can also directly affect the short-chain fatty acid metabolic pathway mediated by intestinal microorganisms that are closely associated with HF (Kho and Lal, 2018; Long et al., 2020). Secondary metabolites of Actinobacteria are an essential source of antibiotics. The increased abundance of Actinobacteria can enhance the production of antibiotics in the gut, disrupting the GM’s homeostasis and exacerbating the progression of associated diseases (Katsuyama, 2019). The increased abundance of Proteobacteria is regarded as a marker of GM disorder and a potential diagnostic indicator of disease (Shin et al., 2015). Proteobacteria can effectively use nitrate produced by inflammatory reactions as electron acceptors for anaerobic respiration; therefore, in an inflammatory environment, Proteobacteria has a proliferation advantage over Firmicutes and Bacteroidetes, which depend on fermentation for growth. Its overgrowth can produce a large amount of endotoxin, aggravate the structural destruction of intestinal epithelial tight junctions, trigger an oxidative stress response and host metabolic disorder, and further aggravate local inflammatory reactions (Shin et al., 2015; Feng et al., 2020). The GM structure in all groups was analyzed at the phylum level. The experimental results showed that the abundance of Firmicutes in the DOX-A to DOX-F, DOX-G and DOX-I groups was lower than that in the CON groups, among which the difference between that in the DOX-I group and CON groups was the most obvious; the abundance of Actinobacteria in the DOX-A to DOX-I groups was higher, among which the difference between the DOX-I group and CON groups was the most obvious; and the abundance of Proteobacteria in the DOX-A to DOX-I groups was higher, in which the difference between the DOX-F group and the CON groups was the most obvious. The ratio of F/B in the DOX-A to DOX-G and DOX-I groups was decreased, among which the difference between the DOX-I and CON groups was the most obvious. The above results are consistent with previous studies. Some studies have pointed out that the abundance of Escherichia/Shigella increases in an HF environment (Pasini et al., 2016; Hayashi et al., 2018). In patients with HF, the abundance of Escherichia/Shigella during the decompensated phase is higher than that during the compensated phase of HF (Emoto et al., 2021). Interestingly, the abundance of Escherichia/Shigella in the large intestine positively correlated with circulating levels of TMAO and indoxyl sulfate (IS) (Tatusov et al., 2003; Kanehisa et al., 2017), and IS, as a microbial metabolite harmful to the cardiovascular system, is closely related to inflammation, endothelial dysfunction, oxidative stress and left ventricular diastolic function (Gao and Liu, 2017). Lactobacillus and Bifidobacterium are the main beneficial bacterial genera in the intestinal tract. Lactobacillus has potent antioxidant activity and can reduce the risk of accumulation of reactive oxygen species (ROS) (Achuthan et al., 2012). It can also inhibit the colonization of pathogenic bacteria in the intestine through competitive exclusion and stimulate the expression of occludin and zonula occludens protein 1 (ZO-1) to improve intestinal barrier function and reduce the inflammatory response (Pourgholam et al., 2016). Bifidobacterium can enhance intestinal epithelial barrier function, inhibit the colonization of pathogenic microorganisms and regulate host immunity to improve the intestinal environment (Zhong et al., 2020). The abundance of Bifidobacterium and Lactobacillus increased, which may have been due to the compensatory effect during the occurrence of HF to balance the effect of harmful bacteria through the increase in the abundance of beneficial bacteria (Kanehisa et al., 2017; Kamo et al., 2017a; Jia et al., 2021). Additional studies have shown that an enhanced abundance of Bacteroides can increase the expression of zonulin protein, thereby improving mucosal integrity (Leiva-Gea et al., 2018; Jia et al., 2021). The GM structure in all groups was analyzed at the genus level. The experimental results showed that the abundance of Escherichia/Shigella was higher in the DOX-A to DOX-I groups than in the CON groups, among which the difference between the DOX-I group and CON groups was the most obvious; the abundance of Lactobacillus was higher in the DOX-B, F, G, H and I groups, among which the differences between the DOX-I group and the CON groups was the most obvious; the abundance of Bifidobacterium was higher in the DOX-A, DOX-C to DOX-I groups, among which the difference between the DOX-F group and the CON groups was the most obvious; and the abundance of Bacteroides was lower in the DOX-A to DOX-I groups, with the most significant decrease in the DOX-C group. These changes in GM observed using this approach were the closest to the changes in the GM of patients with HF observed in previous studies, consistent with the experimental purpose of studying the correlation between HF and the GM.

Notably, rats in the II groups showed similar changes in GM structure to those in the TVI groups. Although studies have shown that the heart is a priority target for DOX toxicity, DOX can also come into direct contact with the gut during II, which subsequently leads to direct injury to the intestinal epithelium and disruption of the physical barriers that separate the intestinal epithelium from the bacteria in the intestinal lumen, such as the mucin barrier, causing disturbances in the GM (Rigby et al., 2016; Ye et al., 2016); thus, it is not possible to exclude the extent to which changes in the GM are affected by the DOX drug itself, which TVI can minimize.The, HE staining results supported this well.

We also used the Tax4Fun function to identify the functional differences within the GM among all groups. The experimental results showed that the functions of the GM in DOX groups were mainly focused on metabolism (amino acid metabolism, glycan biosynthesis and metabolism, enzyme family, biosynthesis of other secondary metabolisms, terpene and polyketide metabolism, etc.), genetic information processing (e.g., folding, sorting and degradation, and translation), cellular processes (transport and catabolism) and human diseases (cancer, cardiovascular diseases), which may influence the development of HF. In particular, only the TVI groups exhibited enrichment in cardiovascular disease pathways.

The shortcomings of this study are as follows. First, due to the small sample size, the influence of individual differences in rats cannot be wholly excluded. The experimental results need to be verified by expanding the sample size in the future. Second, this study did not evaluate different time points of drug administration, and the critical nodes of intestinal microbial structural changes are unclear. Finally, this study did not examine circulating levels of signature GM metabolites (e.g., LPS and TMAO) that are directly associated with adverse cardiovascular events and all-cause mortality. Subsequent experiments can address the above deficiencies and improve the experimental protocol to identify critical bacteria by depleting or introducing specific microbes to lay a foundation for further research.

## Data availability statement

The data presented in the study are deposited in the NCBI(National Center for Biotechnology Information) repository, bio project ID: PRJNA929090, accession number: SRP420114.

## Ethics statement

The animal study was reviewed and approved by the Ethics Committee for the Welfare of Experimental Animals of Shenzhen Hospital of Guangzhou University of Chinese Medicine (Futian), project number: 2021-641.

## Author contributions

TZ, YF, LL and XT designed the study and revised the manuscript. YF, JZ, LM and MW performed the experiments, analyzed the data and wrote the manuscript. XH and SG participated in its design and revised the manuscript. JX and TL performed the statistical analysis. All authors contributed to the article and approved the submitted version.
