# Supplementary figures and images for: Changes in the gut microbiota structure and function in rats with doxorubicin-induced heart failure
Source: Front Cell Infect Microbiol. 2023 Apr 27;13:1135428. doi: 10.3389/fcimb.2023.1135428 (PMC10173310; doi:10.3389/fcimb.2023.1135428)

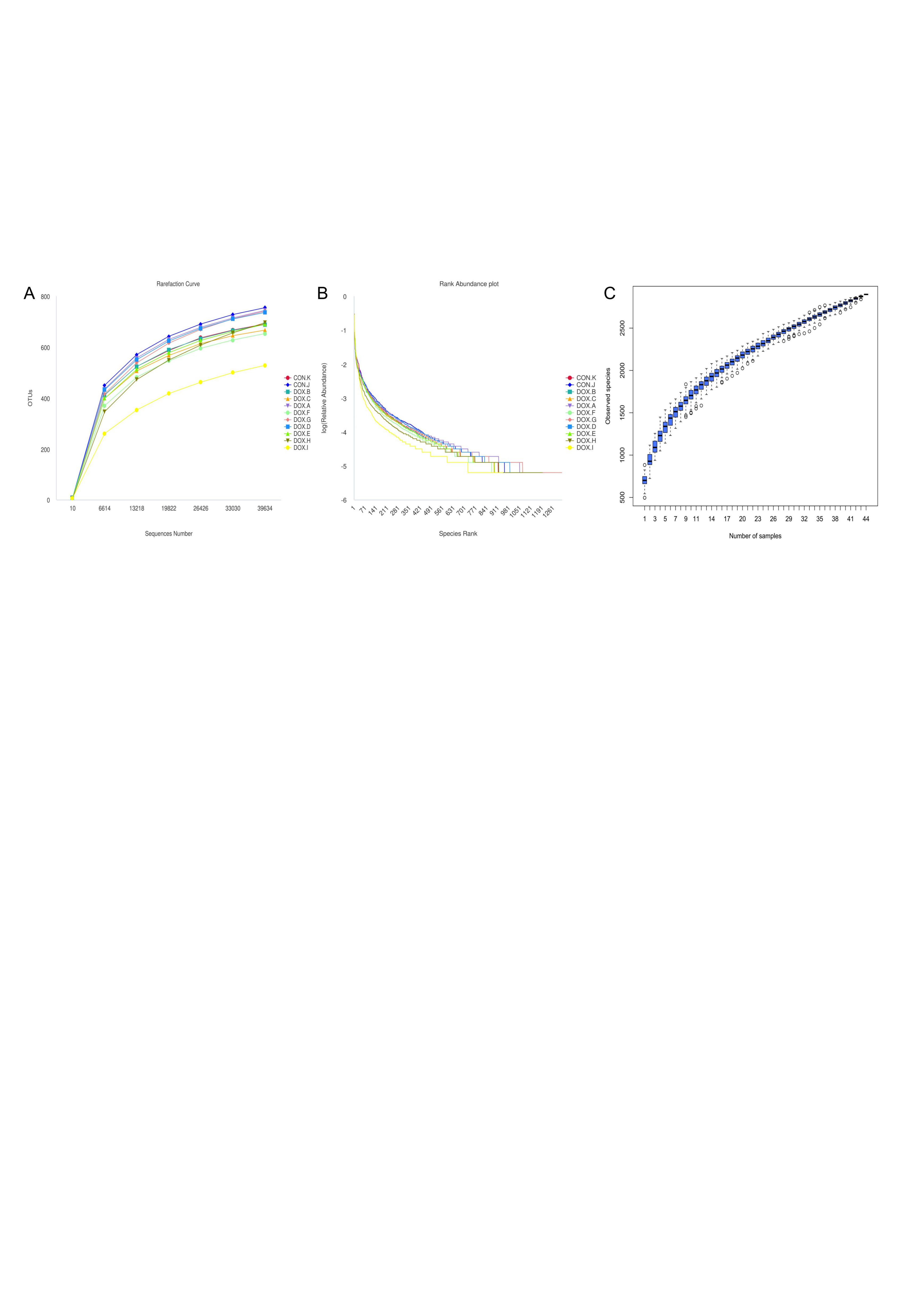

Supplement: Supplementary file 1 [file Image_1.jpg]
