# Supplementary material for: Changes in the gut microbiota structure and function in rats with doxorubicin-induced heart failure
Source: Front Cell Infect Microbiol. 2023 Apr 27;13:1135428. doi: 10.3389/fcimb.2023.1135428 (PMC10173310; doi:10.3389/fcimb.2023.1135428)
Supplement: Supplementary file 2 [file Table_1.docx]

**Supplementary Table S1**

Significant test of community structure difference between groups.

| **Group** | | | **Anosim** | | **MRPP** | | | |
| --- | --- | --- | --- | --- | --- | --- | --- | --- |
|  |  |  | **R** | ***P*** | **Observe Delta** | **Expect Delta** | **A** | ***P*** |
| DOX-A | VS. | CON-J | 0.7917 | 0.030* | 0.4131 | 0.4844 | 0.1470 | 0.041* |
| DOX-B |  |  | 0.9583 | 0.034* | 0.3475 | 0.4421 | 0.2140 | 0.024* |
| DOX-C |  |  | 0.8438 | 0.033* | 0.3988 | 0.4845 | 0.1770 | 0.027* |
| DOX-D |  |  | 0.8333 | 0.028* | 0.3684 | 0.4274 | 0.1380 | 0.026* |
| DOX-E |  |  | 0.9062 | 0.034* | 0.4087 | 0.4825 | 0.1530 | 0.019* |
| DOX-F |  |  | 0.9479 | 0.029* | 0.4063 | 0.5046 | 0.1948 | 0.03* |
| DOX-G | VS. | CON-K | 0.5521 | 0.033* | 0.4538 | 0.5229 | 0.1321 | 0.033* |
| DOX-H |  |  | 0.6354 | 0.030* | 0.4550 | 0.5894 | 0.2281 | 0.023* |
| DOX-I |  |  | 1.0000 | 0.031* | 0.4158 | 0.6251 | 0.3349 | 0.024* |

*Anosim: R-value∈(−1, 1),and R-value>0 indicates significant difference between groups. MRPP: the smaller the observed delta value, the smaller the intra group difference; the larger the expect delta value, the greater the inter group difference. A value > 0 indicates that the between

-groups difference is greater than the within-groups difference.CON, control; DOX, doxorubicin; ^*^*P* < 0.05;^**^*P* <0 .01.
