# Supplementary material for: Changes in the gut microbiota structure and function in rats with doxorubicin-induced heart failure
Source: Front Cell Infect Microbiol. 2023 Apr 27;13:1135428. doi: 10.3389/fcimb.2023.1135428 (PMC10173310; doi:10.3389/fcimb.2023.1135428)
Supplement: Supplementary file 3 [file Table_2.docx]

**Supplementary TABLE 2 |** The significant expression pathways based on Tax4Fun of the gut microbial community at KEGG level 2. CON, control; DOX, doxorubicin; TVI, tail vein injection; II, intraperitoneal injection; KEGG, Kyoto Encyclopedia of Genes and Genomes.

| **Metabolic pathways** | | **TVI** | | **II** | |
| --- | --- | --- | --- | --- | --- |
| Cellular Processes | Transport and catabolism | DOX-A,B,C,D,F |  | DOX-G |  |
|  | Cell motility | N/A | CON-J | N/A | CON-K |
|  | Cellular community-prokaryotes | N/A | CON-J | N/A | N/A |
| Environmental Information Processing | Membrane transport | N/A | CON-J | N/A | N/A |
|  | Signal transduction | N/A | CON-J | N/A | CON-K |
| Genetic Information Processing | Folding,sorting and degradation | DOX-A,B,C,D,E,F | N/A | DOX-G,H,I | N/A |
|  | Translation | DOX-A,B,C,F | N/A | DOX-H,I | N/A |
|  | Replication and repair | N/A | N/A | DOX-I | N/A |
| Metabolism | Metabolism of other amino acids | DOX-A,B,C,E,F | N/A | DOX-G,H | N/A |
|  | Glycan biosynthesis and metabolism | DOX-A,B,E,F | N/A | DOX-G | N/A |
|  | Enzyme families | DOX-B,C,D,F | N/A | DOX-H | N/A |
|  | Biosynthesis of other secondary metabolism | DOX-A,B,D,F | N/A | N/A | N/A |
|  | Metabolism of terpenoids and polyketides | DOX-E,F | N/A | DOX-H,I | N/A |
|  | Lipid metabolism | DOX-A,B,D | N/A | N/A | N/A |
|  | Metabolism of cofactors and vitamins | DOX-A,B | N/A | N/A | N/A |
|  | Carbohydrate metabolism | N/A | N/A | DOX-H,I | N/A |
|  | Nucleotide metabolism | N/A | N/A | DOX-H,I | N/A |
|  | Xenobiotics biodegradation and metabolism | N/A | CON-J | N/A | CON-K |
|  | Amino acid metabolism | N/A | N/A | N/A | CON-K |
| Human Diseases | Cancers | DOX-A,B,C,D,E,F | N/A | N/A | N/A |
|  | Drug resistance | N/A | N/A | DOX-G,H,I | N/A |
|  | Endocrine and metabolic diseases | N/A | CON-J | DOX-I | N/A |
|  | Cardiovascular diseases | DOX-A,B,C,D,F | N/A | N/A | N/A |

**Supplementary TABLE 2 (continued) |** The significant expression pathways based on Tax4Fun of the gut microbial community at KEGG level 2. CON, control; DOX, doxorubicin; TVI, tail vein injection; II, intraperitoneal injection; KEGG, Kyoto Encyclopedia of Genes and Genomes.

| **Metabolic pathways** | | **TVI** | | **II** | |
| --- | --- | --- | --- | --- | --- |
| Organismal Systems | Nervous system | DOX-B,D,F | N/A | N/A | N/A |
|  | Environmental adaptation | N/A | N/A | N/A | CON-K |
|  | Aging | N/A | N/A | DOX-I | N/A |
|  | Immune system | N/A | N/A | N/A | CON-K |
| Unclassified | Cellular processes and signaling | DOX-A,B.D,E,F | N/A | N/A | N/A |
|  | Poorly characterized | N/A | N/A | DOX-G | N/A |
|  | Genetic information processing | N/A | N/A | N/A | CON-K |
|  | Metabolism | N/A | N/A | N/A | CON-K |
